# Metabolic profiling reveals key metabolites regulating adventitious root formation in ancient *Platycladus orientalis* cuttings

**DOI:** 10.3389/fpls.2023.1192371

**Published:** 2023-07-11

**Authors:** Ermei Chang, Wei Guo, Yao Dong, Zirui Jia, Xiulian Zhao, Zeping Jiang, Li Zhang, Jin Zhang, Jianfeng Liu

**Affiliations:** ^1^ State Key Laboratory of Tree Genetics and Breeding, Key Laboratory of Tree Breeding and Cultivation of State Forestry Administration, Research Institute of Forestry, Chinese Academy of Forestry, Beijing, China; ^2^ State Key Laboratory of Subtropical Silviculture, College of Forestry and Biotechnology, Zhejiang A&F University, Hangzhou, Zhejiang, China; ^3^ Taishan Academy of Forestry Sciences, Taian, Shandong, China; ^4^ Key Laboratory of Forest Ecology of National Forestry and Grassland Administration, Environment and Protection, Research Institute of Forest Ecology, Environment and Protection, Chinese Academy of Forestry, Beijing, China; ^5^ College of Agricultural and Biological Engineering, Heze University, Heze, Shandong, China

**Keywords:** ancient tree, lignification, inhibitors, adventitious root formation, wounding

## Abstract

*Platycladus orientalis*, a common horticultural tree species, has an extremely long life span and forms a graceful canopy. Its branches, leaves, and cones have been used in traditional Chinese medicine. However, difficulty in rooting is the main limiting factor for the conservation of germplasm resources. This study shows that the rooting rates and root numbers of cuttings were significantly reduced in ancient *P. orientalis* donors compared to 5-year-old *P. orientalis* donors. The contents of differentially accumulated metabolites (DAMs) in phenylpropanoid (caffeic acid and coniferyl alcohol) and flavonoid biosynthesis (cinnamoyl-CoA and isoliquiritigenin) pathways increased significantly in cuttings propagated from ancient *P. orientalis* donors compared to 5-year-old *P. orientalis* donors during adventitious root (AR) formation. These DAMs may prevent the ancient *P. orientalis* cuttings from rooting, and gradual lignification of callus was one of the main reasons for the failed rooting of ancient *P. orientalis* cuttings. The rooting rates of ancient *P. orientalis* cuttings were improved by wounding the callus to identify wounding-induced rooting-promoting metabolites. After wounding, the contents of DAMs in zeatin (5′-methylthioadenosine, *cis*-zeatin-*O*-glucoside, and adenine) and aminoacyl-tRNA biosynthesis (l-glutamine, l-histidine, l-isoleucine, l-leucine, and l-arginine) pathways increased, which might promote cell division and provided energy for the rooting process. The findings of our study suggest that breaking down the lignification of callus *via* wounding can eventually improve the rooting rates of ancient *P. orientalis* cuttings, which provides a new solution for cuttings of other difficult-to-root horticultural and woody plants.

## 1 Introduction

*Platycladus orientalis* is commonly used for hedges, landscaping, garden courtyards, and bonsais. Ancient *P. orientalis* has the characteristics of wide adaptability and great longevity (Chang et al., 2012). Cutting propagation is a common technique used to preserve excellent *P. orientalis* resources of ancient trees. However, the rooting rates of most tree species (e.g., *Juglans* L., *Olea europaea*, and *Corylus avellana*) decrease sharply with the increase in the tree ages (Cristofori et al., 2010; Porfírio et al., 2016; Song et al., 2021). According to prior research, with the increase in tree age, the rooting rate may be affected by endogenous inhibitors of cuttings, in addition to their physiological and structural changes. For example, phenolics and flavonoids are primary endogenous inhibitors in *Eucalyptus grandis* (Aumond et al., 2017). During the cutting process of ancient *E. grandis*, the expression of *α-tubulin* decreased in inverse proportion to that of secondary metabolites (Abu-Abied et al., 2012). However, there were too few studies focused on the identification of metabolites that inhibit adventitious root (AR) formation in ancient *P. orientalis* cuttings.

As a result of a lack of defense strategies, cuttings propagated from adult trees may resist the invasion of pathogenic bacteria by increasing their phenolic and flavonoid contents. For instance, cadaverine, naringenin, riboflavin, or rutin delayed the rooting of cuttings of *Ilex dumosa* (Luna et al., 2003). The monophenolic compound salicylic acid can inhibit AR formation in stem slices of Malus ‘Jork 9’ (De Klerk et al., 2011). The influence on soluble phenolic content reduced the AR formation, and its oxidation caused the browning and death of the cuttings propagated from adult *Ilex paraguariensis* (Tarragó et al., 2012). Meanwhile, catechol inhibited the rooting of softwood cuttings of *Acer griseum* (Maynard and Bassuk, 1990). Some researchers also surmised that phenolics and flavonoids could promote rooting (Ahammed et al., 2017). For example, the accumulation of high-concentration phenol substances in twig cuttings of *Rosa bourboniana* promoted AR formation (Balakrishnamurthy and Rao, 1988). Exogenous ferulic acid enhanced AR formation and protected indole-3-acetic acid from wounding-induced carboxylation in *Malus pumila* cuttings (De Klerk et al., 2011). Therefore, further studies are necessary to explore the types of endogenous inhibitors for rooting and their influencing mechanisms.

Callus tissues of cuttings are totipotent, being able to regenerate the whole plant body (Steward et al., 1958). Callus was inhibited to develop into root primordium in mature cuttings of *E. grandis* (Abu-Abied et al., 2014). It was more difficult for mature cuttings to form ARs from cell division than juvenile cuttings (Abu-Abied et al., 2012; Abu-Abied et al., 2014). Flavonoids inhibited auxin transport during the AR formation of softwood cuttings of *Camellia sinensis* (Druege et al., 2016; Wang et al., 2022). The research shows that the accumulation of flavonoids further influenced the plant hormone signaling pathway (Peer and Murphy, 2007; da Rocha Correa et al., 2012). The synthesis of flavonoids enhanced the lignification of cuttings at the early stage of AR development in *Morus alba* hardwood cuttings (Tang et al., 2016). The increase in lignin monomers may solidify the callus cell wall and inhibit the root growth of plants (Boerjan et al., 2003). Reduced AR formation was observed in the presence of high content of lignin in cuttings of *Cinnamomum kanehirae* (Cho et al., 2011). In addition, therefore, addressing the problem of callus instead of root primordia may be the key to improving the rooting rate of cuttings of ancient tree.

Plant adaptation to wounding was regulated by the complex interaction of plant hormones, such as jasmonic acid (JA) and ethylene (Liu et al., 2022). For example, according to the results of a metabolomics study, wounding of *Brassica oleracea* primarily caused the production of JA biosynthesis, which was mainly involved in the biosynthesis of aminoacyl-tRNA, amino acids and secondary metabolites, and purine metabolism (Guan et al., 2021). Moreover, wounding broke down the mechanical barrier of lignification of the callus, which induced the production of secondary metabolites around wounds and enhances resistance (Jing et al., 2020). In addition, secondary metabolites participated in the wound recovery of *Quercus ilex* and *C. sinensis* (Sardans et al., 2014; Zhao et al., 2020). The contents of coumarin and fatty acyl increased in wounded *Pastinaca sativa* leaves (Galati et al., 2019). The contents of flavonoids, flavonol glycosides, and isorhamnetin also exhibited significant increases in *Ginkgo biloba* leaves after pruning (Cao et al., 2022). However, the relevant metabolites during wounding-induced AR formation in cuttings propagated from ancient *P. orientalis*.

*P. orientalis* is extremely long-lived and often planted in temples, mausoleums, and horticultural fields (Chang et al., 2012). Herein, given the difficulty in rooting the ancient *P. orientalis* cuttings (Wei et al., 2020), the present study employed metabonomic and proteomic sequencing at the critical stages of 5-, 100-, and 700-year-old *P. orientalis* cuttings to identify the endogenous inhibitors of AR formation. It is completely unknown what prevents the cuttings from taking root or whether rooting of the cuttings could be promoted through wounding. Meanwhile, the mechanical barriers were broken down by wounding to identify key metabolites related to defense responses and regeneration during AR formation. This study is expected to provide a theoretical basis for improving the rooting rate and shortening the rooting time of ancient *P. orientalis* cuttings and other difficult-to-root horticultural plants.

## 2 Materials and methods

### 2.1 Cutting materials

Age groups including nine of 5-, 100-, and 700-year-old *P. orientalis* donors were selected in Beijing Botanical Garden to collect strong and pest-free branches that were 3–5 m above the ground. They were cut into 10–15-cm cuttings at the base 1.0–1.5 cm from buds. The cuttings were dipped in sodium lignosulfonate:NAA : IBA = 1:1:1 at the concentration of 1,000 ppm for 1 min, with 25 cuttings in each treatment and three repeats for each treatment. The cutting experiment was carried out under full sunshine and automatic spray at the Chinese Academy of Forestry.

### 2.2 Adventitious root forming process materials

Stage 1 (S1) was the *P. orientalis* cuttings have just been taken. Stage 2 (S2) was when the white or red callus began to grow at the cut site of the cuttings 45 days after cutting. Stage 3 (S3) was when the emergence of AR tips from the callus was evident 90 days after cutting. A scalpel was used to peel off the xylem at a distance of 0.5 cm from the base of the cuttings of S1, S2, and S3, and the remaining parts were then put into liquid nitrogen as metabolome and proteome materials.

### 2.3 Wounding treatments

After the culture of the cuttings propagated from 100-year-old *P. orientalis* for 30 days, the callus was incised, and the non-incised callus was used as control. The wounded cuttings were dipped in sodium lignosulfonate:NAA : IBA = 1:1:1 at the concentration of 1,000 ppm for 1 min, with 25 cuttings in each treatment and three repeats for each treatment. After culturing for 40 days, the rooting rates and root numbers were counted, and 0.5-cm branches (without xylem) at the wounded (ck, w-s1, and w-s2) and non-wounded (ck, nw-s1, and nw-s2) cutting base were collected at 0, 20, and 40 days and then put into liquid nitrogen as metabolome and proteome materials.

### 2.4 Metabolite extraction

A powder sample (50 mg) was collected in a 2-ml centrifuge tube containing grinding beads. Metabolites were extracted using 400 μl of extracting solution containing 0.02 mg/ml internal standard (Vmethanol : Vwater = 4:1, with 0.02 mg/ml of l-2-chlorophenylalanine as the internal standard). The sample solution was ground at −10°C and 50 Hz for 6 min in a frozen tissue grinder, and then extraction was performed by low-temperature ultrasound at 5°C and 40 kHz for 30 min. Afterward, the sample was placed at −20°C for 30 min, centrifuged at 13,000 *g* for 15 min at 4°C, and transferred into a tube for liquid chromatography-mass spectrometry (LC-MS) detection and analysis. All metabolites of the same volume were collected and mixed to prepare quality control (QC) samples. One QC was inserted into every 10 samples to investigate the repeatability of the whole analysis.

### 2.5 LC-MS/MS analysis

LC-MS analyses were performed using a UHPLC-Q Exactive HF-X system (1290, Agilent Technologies, Shanghai, China) with a UPLC HSS T3 column (100 mm × 2.1 mm i.d., 1.8 µm) coupled to Q Extractive unit (Orbitrap MS, Thermo, Waltham, MA, USA). Mobile phase A was 95% water + 5% acetonitrile (containing 0.1% formic acid), and mobile phase B was 47.5% acetonitrile + 47.5% isopropanol + 5% water (containing 0.1% formic acid). The gradient elution procedure of the mobile phases is according to Xie et al. (2019). The mass spectrum (MS) signals of the sample were scanned in positive and negative ion modes, with a mass range of 70–1,050 *m*/*z*. The sheath gas flow rate was set at 50 psi, the auxiliary gas flow rate at 13 psi, the auxiliary gas heating temperature at 425°C, the ion spray voltage in the positive mode at 3,500 V, the ion spray voltage in the negative mode at −3,500 V, the ion-transfer tube temperature at 325°C, and the normalized collision energy at 20–40–60 V cyclic collision energy. The resolution of primary MS was 60,000, and that of secondary MS was 7,500. The data were collected using data-dependent acquisition (DDA) mode.

### 2.6 LC/MS data preprocessing

After analysis, LC-MS raw data were imported into metabonomic processing software progenesis for baseline filtering, peak recognition, integration, retention time correction, and peak alignment. Finally, a data matrix of retention time, mass-to-charge ratio, and peak intensity was obtained. In addition, the MS and tandem MS data were matched with the public metabonomic databases HMDB (http://www.hmdb.ca/) and Metlin (https://metlin.scripps.edu/), and the self-built database of Majorbio to obtain the metabolite information.

The matrix data after the database search were uploaded to the Majorbio Biological Cloud Platform (https://cloud.majorbio.com) for data analysis. First, data preprocessing was conducted. The missing values of the data matrix were removed using the 80% rule; that is, more than 80% of the non-zero variables in at least one group of samples were retained, and then vacant values were filled in (with the minimum value of the original matrix). To reduce the errors caused by sample preparation and instrument instability, the response intensity of the MS peaks was normalized using the sumNormalizer to obtain the normalized data matrix. Additionally, the variables with relative standard deviation (RSD) of QC samples >30% were deleted, and logarithmic processing with log10 was performed to obtain the final data matrix for subsequent analysis.The differences in the preprocessed matrix file were analyzed. The R package ropls performed principal component analysis (PCA) and orthogonal least partial squares discriminant analysis (OPLS-DA). The stability of the model was evaluated by seven cycles of interactive verification. Moreover, Student’s *t*-test and the fold-change analysis were carried out. Differential metabolites were selected based on the variable weight value (variable importance in projection (VIP)) obtained from the OPLS-DA model and the *p*-value from Student’s *t*-test. The metabolites with fold change (FC) >1.5 or <0.67, VIP > 1, and *p* < 0.05 were significant differential metabolites. The differential metabolites were annotated by the Kyoto Encyclopedia of Genes and Genomes (KEGG) database (https://www.kegg.jp/kegg/pathway.html) to obtain the pathways involved in the differential metabolites. Pathway enrichment analysis was conducted using the Python software package scipy.stats, and the biological pathway most relevant to the experimental treatment was obtained by Fisher’s exact test.

### 2.7 Proteome sequencing

For proteome analysis, iTRAQ technology was used to obtain raw data, Uniprot database was used for searching, *t*.test function in R language was used to calculate the *p*-value of the significant difference between samples, and at the same time, the fold of difference between groups was calculated. The screening criteria for significantly differentially expressed proteins (DEPs) were as follows: upregulated proteins with *p* < 0.05 and FC > 1.2, and downregulated proteins with *p* < 0.05 and FC < 0.83. Then, KEGG pathway analysis was performed for these DEPs.

### 2.8 Correlation analysis between DEPs and DAMs

The known proteins related to the biosynthetic pathways of phenolics and flavonoids were found in proteomics data during AR formation of the cuttings propagated from *P. orientalis* donors at different ages. Meanwhile, relative contents of phenolics and flavonoids were obtained from metabonomics data during AR formation of the cuttings propagated from *P. orientalis* donors at different ages. The correlation between proteins and metabolites was calculated as Pearson’s correlation coefficients. Correlation pairs were deemed statistically significant when the absolute value of Pearson’s correlation coeffificient (PCC) >0.9 and p-value<0.01.

### 2.9 Determination of physiological changes

In order to investigate physiological changes, endogenous phenolics, flavonoids, total soluble sugar, soluble protein, and lignin contents were quantified by using an assay kit (Cominbio, Suzhou, China) according to the methods of Qian et al. and Du et al. (Du et al., 2019; Wei et al., 2020).

For lignin staining, transverse sections were dipped in 1% phloroglucinol and 12% HCl for 10 min, shifted in an acidic solution (10% H_2_SO_4_ and 75% glycerol), and observed under bright-field Leica M205 FA microscope (Leica Microsystems, Wetzlar, Germany). Indole-3-acetic acid (IAA) and zeatin were extracted using isopropanol/water/hydrochloric acid and quantified by using a UPLC-MS/MS platform (Agilent 1290 Infinity II UPLC system equipped with the AB Sciex QTrap 6500+ mass spectrometer) and calibrated by using inositol as an internal control.

## 3 Result

### 3.1 Effect of tree age on AR formation of *P. orientalis* cuttings

**Figure 1:**
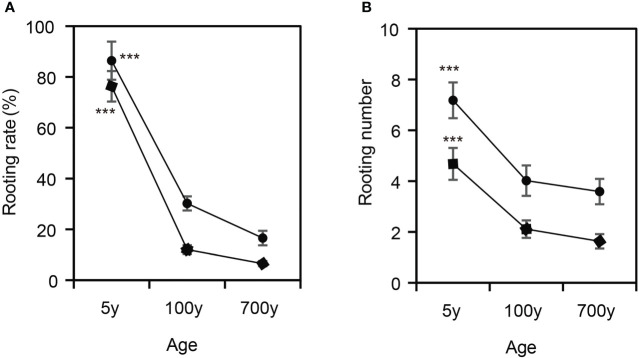
Characteristics of adventitious root (AR) formation of cuttings propagated from *Platycladus orientalis* donors at different ages. **(A)** The rooting rates and **(B)** the root numbers of ARs in cuttings propagated from 5-, 100-, and 700-year-old *P. orientalis* donors. The bars indicate the means ± SDs (n = 9); *p*-values are indicated as ***p < 0.001. Statistical significance was determined by one-way ANOVA with Tukey’s *post-hoc* test.

Observation of the AR formation process of cuttings propagated from *P. orientalis* donors at different ages revealed that the rooting rates and root numbers of cuttings propagated from 5-year-old *P. orientalis* donors (control) were 78.14% and 7.17, respectively, which were significantly higher than those of 100- and 700-year-old *P. orientalis* donors (*p* < 0.05) (**Figure 1**). However, no significant difference was observed in the rooting rates and root numbers of the cuttings propagated from 100- and 700-year-old *P. orientalis* donors (*p* > 0.05). These results suggest that the rooting rates of cuttings may decrease significantly in the age of ancient trees.

### 3.2 Comparison of the number of DAMs during AR formation of *P. orientalis* cuttings

**Figure 2:**
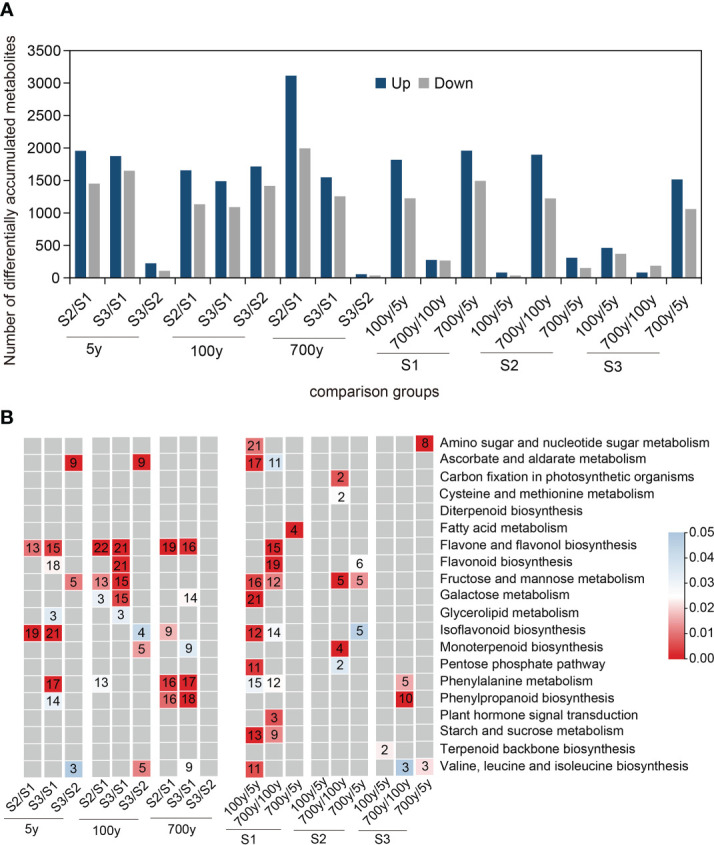
Qualitative and comparative analyses of differentially accumulated metabolites (DAMs) during adventitious root (AR) formation in cuttings propagated from *Platycladus orientalis* donors at different ages. **(A)** Comparison of the number of containing up- and downregulated metabolites in the cuttings propagated from *P. orientalis* donors at different ages. Example of abbreviations: S2/S1 means the ratio of S2 to S1. **(B)** Summary of significantly enriched (*p* < 0.05) pathway terms associated with DAMs identified during AR formation in cuttings propagated from *P. orientalis* donors at different ages. Red color panels illustrate the significance level of enrichment. Non-significant values are typed in gray. Example of abbreviations: 100y/5y means the ratio of 100 to 5 years.

Ultraperformance liquid chromatography–tandem mass spectrometry sequencing was performed to uncover relevant metabolites during the AR formation of *P. orientalis* cuttings. The relatively clustered distribution within each group implied good repeatability, and the evident difference in metabolites among groups suggested biological differences among samples. A total of 8,623 anionic and 9,231 cationic metabolites were detected in this experiment. Further comparison of the numbers of differentially accumulated metabolites (DAMs) among groups revealed the obvious differences among S1, S2, and S3 of the cuttings propagated from 5-, 100-, and 700-year-old *P. orientalis* donors (**Figure 2A**). The numbers of DAMs at S3/S1 and S2/S1 were less than those at S3/S2, which indicated that the numbers of DAMs in cuttings increased with the AR formation process. In addition, the numbers of DAMs in cuttings propagated from 100- and 700-year-old *P. orientalis* donors were the least at S2 and S3, respectively, which denoted a similarity in their AR formation process.

### 3.3 Comparison of the type of DAMs during AR formation of *P. orientalis* cuttings

To explore the types of DAMs in different stages of AR formation, we performed KEGG pathway enrichment analysis of significantly upregulated DAMs in these groups. The upregulated metabolic pathways (flavone and flavonol biosynthesis pathway and phenylalanine metabolism biosynthesis pathway) were significantly enriched at S2/S1 and S3/S1 of cuttings propagated from 100- and 700-year-old *P. orientalis* donors (*p* < 0.05) (**Figure 2B**). The flavonoid biosynthesis pathway and fructose and mannose metabolism pathway were enriched at S3/S1 of cuttings propagated from 5- and 100-year-old *P. orientalis* donors, which suggested that in addition to defense response, energy synthesis is required for AR formation. Notably, a significant accumulation of phenolics occurred in the phenylalanine metabolism biosynthesis pathway at S2 and S3 of cuttings propagated from 100-year-old *P. orientalis* donors, which indicated that the syntheses of secondary metabolites and stress-resistant substances were possible to inhibit the AR formation of ancient trees.

### 3.4 Discovery and identification of the type of DAMs based on combined metabolomic and proteomic analyses

**Figure 3:**
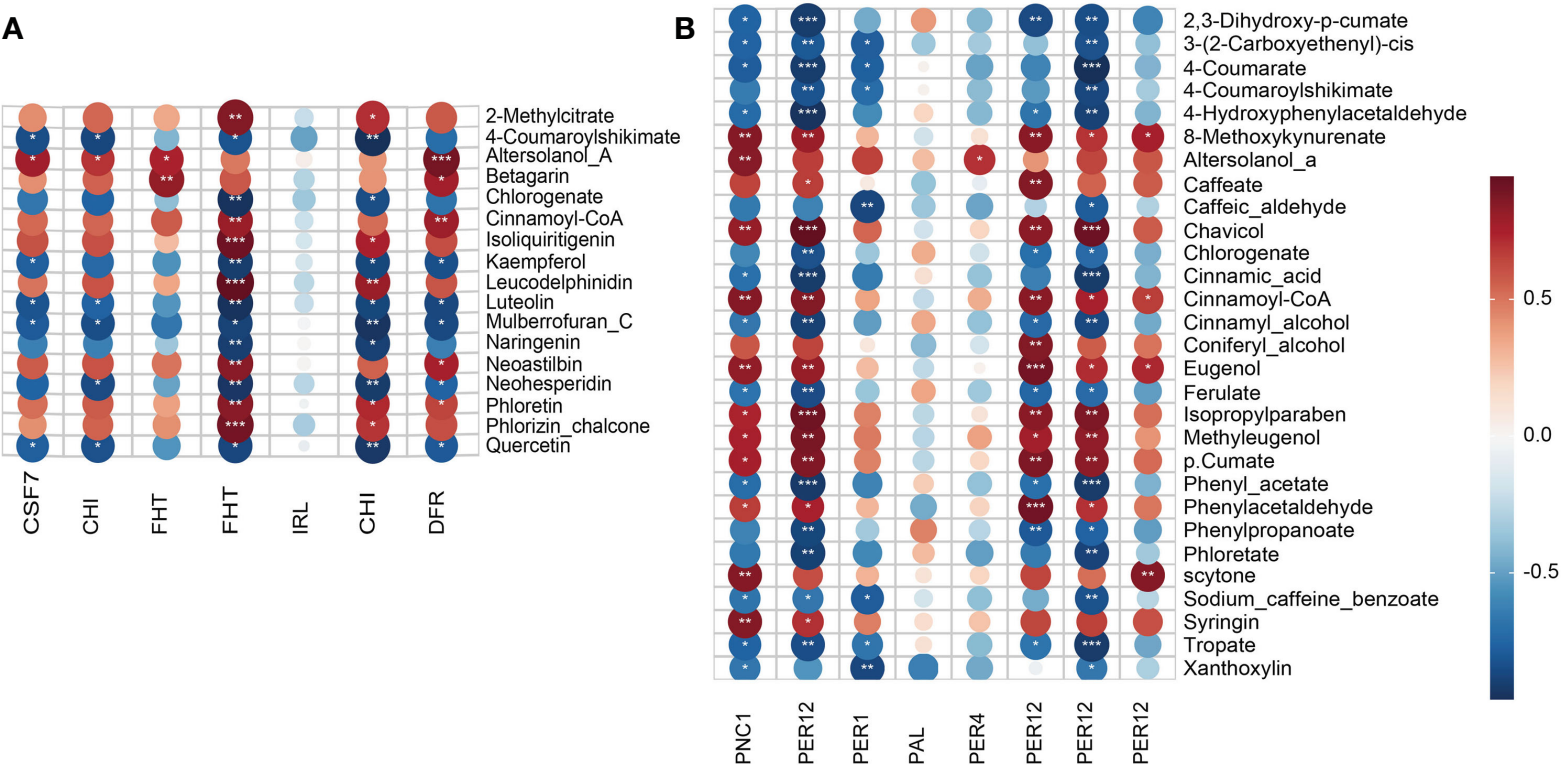
Correlation plot acting in the flavonoid **(A)** and lignin **(B)** biosynthesis pathways during adventitious root (AR) formation in cuttings propagated from *Platycladus orientalis* donors at different ages. Pearson’s correlation coefficient (PCC) between flavonoid and phenylpropanoid biosynthesis pathway differentially accumulated metabolites (DAMs) versus the expression levels of differentially expressed proteins (DEPs). The PCC and significances (*p < 0.05) are presented in a symmetric matrix, and a heatmap is used to indicate the strength of correlation among the variables. Red and blue squares indicate positive and negative correlations, respectively. *p*-Values of one-way ANOVA of data are indicated as follows: *p < 0.05, **p < 0.01, and ***p < 0.001. Protein name abbreviations: CSF7, chalcone synthase 7; CHI, chalcone-flavonone isomerase; FHT, naringenin,2-oxoglutarate 3-dioxygenase; IRL, isoflavone reductase-like protein; CHI, chalcone–flavonone isomerase; DFR, bifunctional dihydroflavonol 4-reductase; PNC1, cationic peroxidase SPC4; PER12, peroxidase 12; PER1, 1-Cys peroxiredoxin A; PAL, phenylalanine ammonia-lyase; PER4, peroxidase 4; PER12, peroxidase 12.

**Figure 4:**
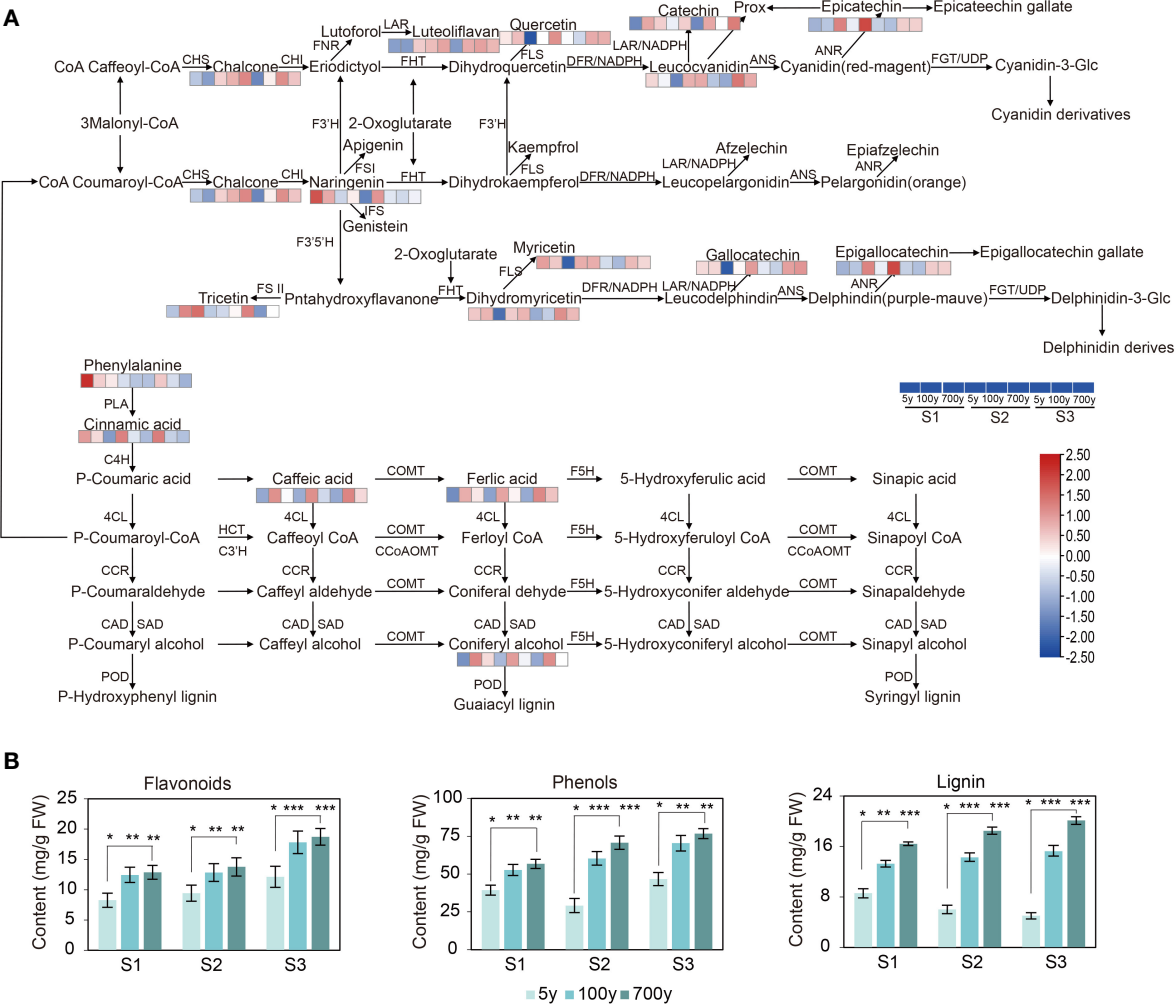
**(A)** Expression patterns of differentially accumulated metabolites (DAMs) involved in the flavonoid, phenylpropanoid, and lignin biosynthesis pathways of cuttings propagated from *Platycladus orientalis* donors at different ages and for the comparisons of different developmental stages. Heatmap of DAMs involved in the schematic flavonoid, phenylpropanoid, and lignin biosynthesis pathways. The fold-change values were normalized using the LogNormalize method. The colors indicate the expression levels (FC) of the DAMs during adventitious root (AR) formation in cuttings propagated from *P. orientalis* donors at different ages. PAL, phenylalanine ammonia-lyase; C4H, cinnamate 4-hydroxylase; 4CL, 4-coumarate: CoA ligase; HCT, hydroxycinnamoyl-CoA shikimate/quinate transferase; C3H, coumarate 3-hydroxylase; CCoAOMT, caffeoyl-CoA-*O*-methyltransferase; CCR, cinnamoyl CoA reductase; F5H, ferulate 5-hydroxylase; COMT, caffeic acid *O*-methyltransferase; CAD, cinnamyl alcohol dehydrogenase; SAD, sinapyl alcohol dehydrogenase; PER, peroxidase; LAC, laccase; CHS, chalcone synthase; CHI, chalcone isomerase; F3H (FHT), flavanone 3-hydroxylase; F3′5′H, flavonoid 3′5′-hydroxylase; FLS, flavonol synthase; DFR, dihydroflavonol 4-reductase; LDOX, leucoanthocyanidin dioxygenase; GST, glutathione *S*-transferase; ANR, anthocyanidin reductase; ANS, anthocyanidin synthase; UFGT, anthocyanidin 3-*O*-glucosyltransferase; ANR, anthocyanidin reductase; ANS, anthocyanidin synthase; LAR, leucoanthocyanidin reductase. **(B)** Flavonoids, phenolics, and lignin abundance during AR formation of *P. orientalis* cuttings. The bars indicate the means ± SDs (n = 9); *p*-values are indicated as follows: *p < 0.05; **p < 0.01; ***p < 0.001. Statistical significance was determined by one-way ANOVA with Tukey’s *post-hoc* test.

To discover the key DAMs involved in the phenylpropanoid and flavonoid biosynthesis pathway that inhibit the AR formation of cuttings propagated from ancient trees (**Tables S1, S2**), PCC analysis was conducted on DAMs and DEPs (**Tables S3, S4**). As for the phenylpropanoid biosynthesis pathway, 450 pairs of correlations, including 216 and 234 pairs of positive and negative correlations, were observed (**Figure 3A**; **Tables S5, S6**). Meanwhile, for the flavonoid biosynthesis pathway, 420 pairs of correlations, including 222 pairs of positive correlations and 198 pairs of negative correlations, were generated. With |PCC| > 0.8 and *p* < 0.01 as the criteria, 13 DAMs (such as coniferyl alcohol, cinnamoyl-CoA, and caffeic acid) and 16 DAMs (such as cinnamic acid, caffeic aldehyde, and 4-coumarate) were screened with strong positive and strong negative correlations in the phenylpropanoid biosynthesis pathway, respectively (**Figure 3B**). Meanwhile, nine DAMs such as phloretin, cinnamoyl-CoA, and isoliquiritigenin had strong negative correlations, and eight DAMs such as quercetin, naringenin, and chlorogenic acid had strong negative correlations in the flavonoid biosynthesis pathway. In addition, the upregulated expression of peroxidase (PER) related to the phenylpropanoid biosynthesis pathway promoted the accumulation of lignin synthesis metabolites (**Figure 4A**). Moreover, the synthesis pathways of phenylpropane, phenolics, and flavonoids were the main pathways that formed lignin.

### 3.5 Comparison of internal substances during AR formation of *P. orientalis* cuttings at different tree ages

In view of the aforementioned research, the ancient *P. orientalis* produced a large number of secondary metabolites during the cutting process. The contents of phenolics, flavonoids, and lignin increased with the increase in tree age of *P. orientalis* and during AR formation. The cuttings propagated from 5-year-old *P. orientalis* donors contained significantly higher auxin content than the 100- and 700-year-old *P. orientalis* donors (*p* < 0.05) (**Figure 4B**). A negative correlation existed between secondary metabolites. In addition, no significant difference was observed at the same stage of cuttings propagated from 100- and 700-year-old *P. orientalis* donors, which suggested small differences in ancient *P. orientalis* cuttings.

### 3.6 Wounding improved the rooting rate of 100-year-old *P. orientalis* donors

**Figure 5:**
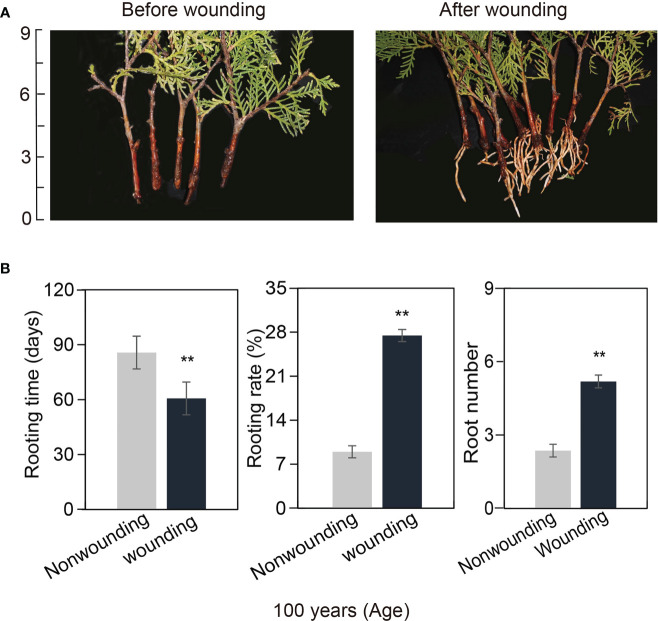
Wound-induced adventitious root (AR) formation in 100-year-old *Platycladus orientalis* cuttings. **(A)** The formation of AR morphology before and after wounding. Scale bar in centimeters. **(B)** The rooting rates, root numbers, and rooting time of ARs in 0, 20, and 40 days after wounding and non-wounding. The bars indicate the means ± SDs (n = 9); *p*-values are indicated as follows: **p < 0.01. Statistical significance was determined by one-way ANOVA with Tukey’s *post-hoc* test.

To promote AR formation, we employed wounding to break down the mechanical barrier of lignification of callus in cuttings propagated from ancient *P. orientalis* donors. After wounding, the rooting rates and rooting numbers of cuttings propagated from 100-year-old *P. orientalis* donors were 11.1% and 2.31, respectively, which were significantly higher than those of cuttings without wounding, accompanied by a significantly shortened rooting time (*p* < 0.05) (**Figure 5**). These data suggest that wounding significantly improved the rooting outcome of ancient *P. orientalis* cuttings.

### 3.7 Analysis of metabolic pathway during wounding-promoted AR formation

**Figure 6:**
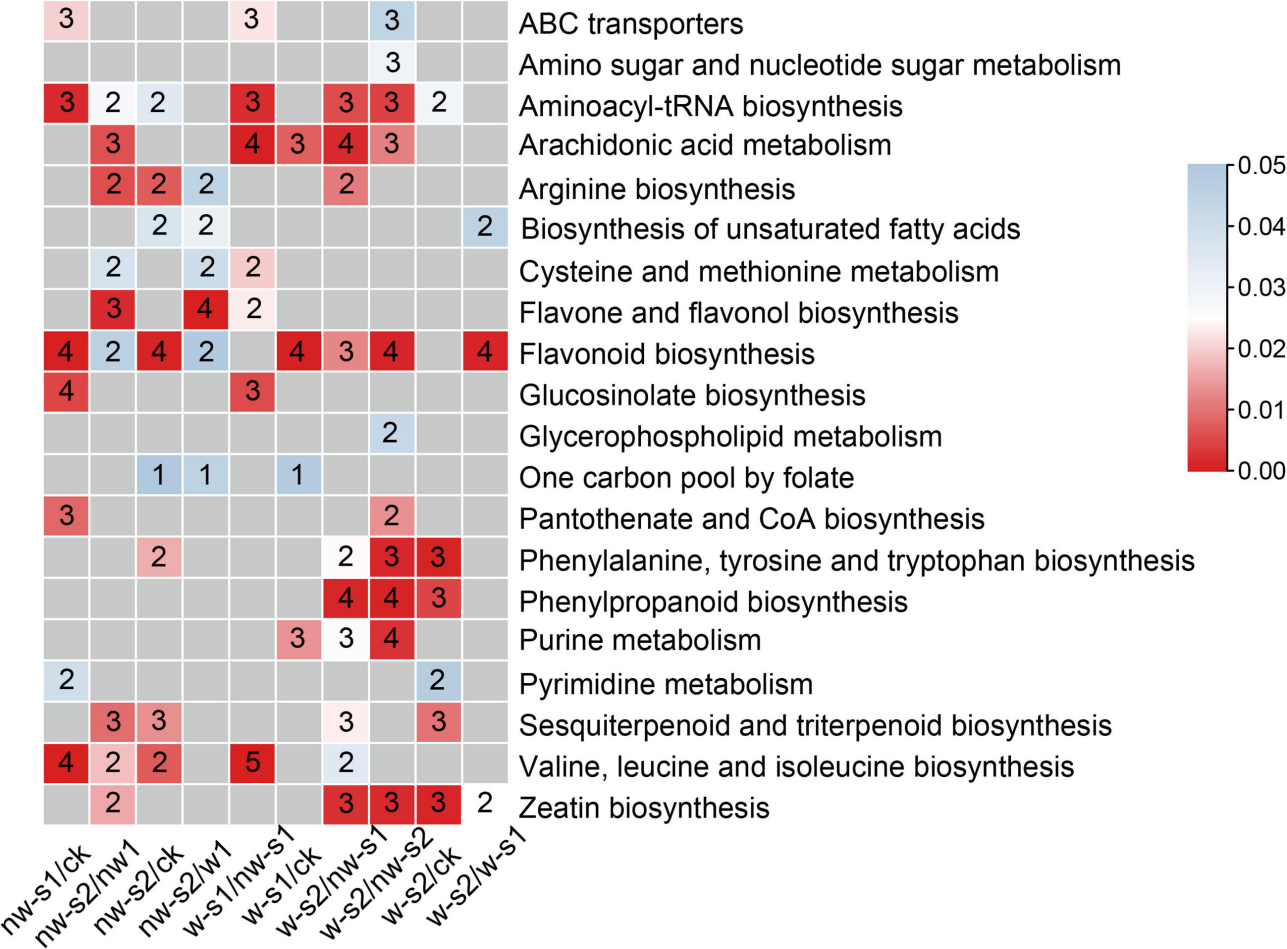
Identification of differentially accumulated metabolites (DAMs) of wound-induced adventitious root (AR) formation in 100-year-old *Platycladus orientalis* cuttings. Summary of significantly enriched (*p* < 0.05) pathway terms associated with DAMs. Red square represents pathways that are significantly high accumulated; the number in the square indicates the metabolite’s number of pathways. Non-significant values are typed in gray. Color panels illustrate the significance level of enrichment.

Our study further identified the key metabolites of cuttings in the process of wounding-promoted AR formation. The screening of significant DAMs that were upregulated in w-s2 than in nw-s1 and nw-s2 was mainly enriched during the biosynthesis of zeatin, aminoacyl-tRNA, and phenylpropanoid at *p* < 0.05 (**Figure 6**). This result indicated the promoted cell division and energy accumulation in AR formation after wounding. Meanwhile, DAMs that were upregulated in w-s1 compared with nw-s1 were enriched significantly in aminoacyl-tRNA biosynthesis and arachidonic acid metabolism pathway and thus served as a material basis for AR formation.

### 3.8 Analysis of key DAMs during wounding-promoted AR formation

**Figure 7:**
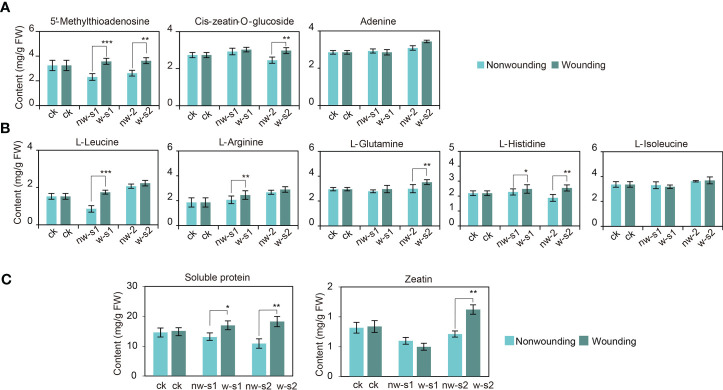
Differentially accumulated metabolite (DAM) accumulation during adventitious root (AR) formation of *Platycladus orientalis* cuttings. **(A)** DAMs of zeatin and **(B)** aminoacyl-tRNA biosynthesis pathways accumulate during the AR formation of cuttings propagated from *P. orientalis* donors at different ages after wounding. **(C)** The changes of zeatin and soluble protein content during AR formation in cuttings propagated from *P. orientalis* donors at different ages. The bars indicate the means ± SDs (n = 9); *p*-values are indicated as follows: *p < 0.05; **p < 0.01; ***p < 0.001. Statistical significance was determined by one-way ANOVA with Tukey’s *post-hoc* test.

Our subsequent analysis clarified changes in the contents of key metabolites after wounding. The contents of 5′-methylthioadenosine, *cis*-zeatin-*O*-glucoside, and adenine in zeatin biosynthesis gradually increased after wounding (**Figure 7A**). Thus, wounding reactivated and promoted cell division, which regulated the differentiation and growth of various cells (Antoniadi et al., 2022; Sun et al., 2023). Metabolites, such as l-glutamine, l-histidine, l-isoleucine, l-leucine, and l-arginine, are involved in aminoacyl-tRNA biosynthesis and are sources of carbon skeleton for basic metabolite synthesis on wounding response (**Figure 7B**) (Guan et al., 2021). Meanwhile, after the first wounding, secondary metabolic activities were enhanced, with increased contents of phenylalanine, phenolics, flavonoids, and other secondary metabolites. Therefore, the stress resistance of the callus improved after wounding.

### 3.9 Comparison of key substances during wounding-promoted AR formation

Finally, this study verified relevant DAMs during wounding-promoted AR formation. The contents of zeatin and soluble proteins increased significantly after wounding and reached the peak at w-s2 (*p* < 0.05) (**Figure 7C**). Hence, zeatin may promote cell redivision around the wound, and soluble proteins regulate osmotic stress and provide a carbon source for cell growth. In addition, the contents of phenolics and flavonoids increased after wounding, which indicated improved free radical scavenging activity and damage resistance.

## 4 Discussion

The present study was designed to clarify the reasons for the decreased rooting rate of cuttings propagated from ancient *P. orientalis* with the increase in the age of the donor tree. Metabolomics data showed that phenylpropanoid and flavonoid pathways were significantly enriched at S2 and S3 in ancient *P. orientalis* donors. The cuttings propagated from 100- and 700-year-old *P. orientalis* donors exhibited significantly lowered rooting rates and higher content of flavonoids, phenolics, and lignin, similar to the findings of previous reports stating that phenolics inhibited AR formation in *I. paraguariensis* and *E. grandis* cuttings (Abu-Abied et al., 2012; Tarragó et al., 2012). Lignin was the final product of the phenylpropanoid pathway (Wang et al., 2016), and prior research reported a decreased AR formation in the presence of high content of lignin in *C. kanehirae* cuttings (Cho et al., 2011). The observation was confirmed by histological analysis that revealed the lignification of callus at S3 in cuttings propagated from ancient *P. orientalis* donors. As evidenced by previous data, lignification is the major inhibitor of the AR formation of plants (Zhao, 2016; Singh et al., 2019). Lignification of callus can be considered not only a barrier against microbial infections but also a highly important inhibitor of AR formation of cuttings propagated from ancient *P. orientalis*.

The incorporation of lignin into the cell walls resulted in structural rigidity, therefore providing barriers to pathogen infection (Lee et al., 2019). Coniferyl alcohol, cinnamoyl-CoA, and caffeic acid contents increased during AR formation in cuttings propagated from 100- and 700-year-old donors, and all of them were intermediate products during lignin formation. Coniferyl and cinnamyl alcohols were important substances in the flavonoid metabolism pathway and participated in lignification (Ahmed et al., 2015; Yang et al., 2021). Therefore, flavonoids and phenolics may be endogenous inhibitors hindering the AR formation of tree cuttings. However, flavonoids promoted rooting in *Cucumis sativus* cuttings (Ahammed et al., 2017). The contents of chlorogenic acid, quercetin, and ferulic acid decreased in ancient trees, and exogenous quercetin enhanced AR formation in cuttings propagated from *I. paraguariensis* (Tarragó et al., 2005). *p*-Coumaric, caffeic, and chlorogenic acids enhanced rooting when supplied alone and increased the effect of auxin applications (Jarvis and McNaughton, 1986), but the role of the substances including coniferyl alcohol, cinnamoyl-CoA, and caffeic acid still needs further verification in AR formation of tree cuttings.

Wounding-induced AR regeneration is a complex regulatory network and an adaptive mechanism involving signaling and material metabolism (Matosevich et al., 2020). Significantly increased rooting rates and root numbers of the callus were detected after wounding of cuttings propagated from ancient *P. orientalis* donors, and such finding was similar to the results of improving the biological yield of tree crops by pruning (Cao et al., 2022). Similar to the results of metabolic pathways in cuttings after the rejuvenation of walnut, the contents of zeatin, amino acid synthetics, and other substances in the callus increased after wounding, which provided a basis for cell differentiation and growth (Song et al., 2021). Zeatin is an important hormone controlling plant growth and development, in which 5′-methylthioadenosine (MTA) is a key metabolite of the Met cycle (Jin et al., 2013; Antoniadi et al., 2022). For instance, MTA nucleosidase activity was the highest during the fruit development period of *Lycopersicon esculentum* (Kushad et al., 1985). Meanwhile, the accumulation of *cis*-zeatin-*O*-glucoside promoted meristem differentiation and increased the number of pods of *Arabidopsis thaliana* and apical flower-bud differentiation of rape (Bürstenbinder et al., 2010; Tarkowská et al., 2019). Moreover, exogenous adenine promoted the regeneration of explants of *Cosmos bipinnatus*, *Crassocephalum crepidioides*, and *Nicotiana tabacum* (Sakpere et al., 2015; Jaberi et al., 2018; Wang et al., 2023). Furthermore, proteins participate in catabolism and are decomposed into carbon, energy, and nitrogen sources. In our study, the contents of substances related to aminoacyl-tRNA biosynthesis increased in the callus of cuttings propagated from ancient *P. orientalis* donors after wounding, consistent with the increasing trend of aminoacyl-tRNA biosynthesis in the wound of *B. oleracea* (Guan et al., 2021). l-Glutamine and l-histidine are both derived from α-ketoglutarate, which promoted plant somatic embryogenesis (Dahrendorf et al., 2018). In another study, after exogenous application of l-isoleucine and l-leucine, the growth of *Lactuca sativa* seedlings was unaffected by salt stress (Mills, 2014). The role of zeatin and energy in the AR formation of tree cuttings has hardly been studied, so further research is needed.

Wounding induced plant regeneration and defense response and broke down the mechanical barrier of callus lignification (Jing et al., 2020). Synthesis of secondary metabolites further enhanced the resistance to pathogenic bacteria. As discovered in our study, after wounding, phenolics, flavonoids (quercetin and isoeugenol), and other substances accumulated in ancient *P. orientalis* cuttings can effectively resist oxidation and enhance the tolerance to various stressors (Mignolli et al., 2017). The findings were consistent with the significantly increased contents of phenolics and carbohydrates in healthy leaves of *Diospyros melanoxylon* after pruning (Mehta et al., 2020). Nevertheless, the balance between zeatin and secondary metabolites in promoting AR formation and lignification after wounding deserves further exploration.

## 5 Conclusion

**Figure 8:**
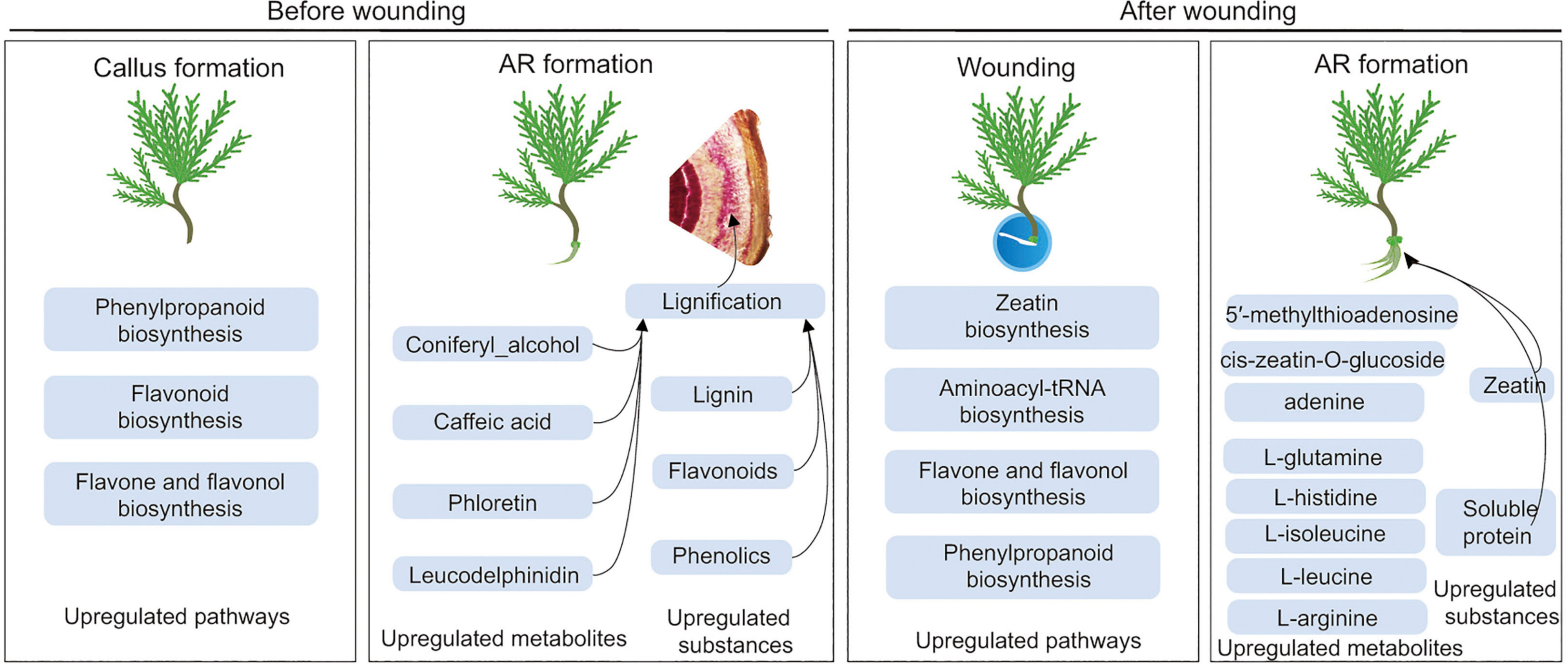
Regulatory model of differential metabolites involved in the significantly enriched metabolic pathways related to inhibitors and promoters during adventitious root (AR) formation in cuttings propagated from *Platycladus orientalis* donors at different ages.

In view of the difficult rooting of ancient *P. orientalis* cuttings, the present study discovered that phenolic acids and flavonoids may inhibit the AR formation of ancient *P. orientalis* cuttings. Phenolic acids and flavonoids are key substances for the lignification of callus, and they can further inhibit AR formation (**Figure 8**). This study realized the significant improvement in the AR formation of ancient *P. orientalis* cuttings by wounding. Moreover, zeatin and energy substances can promote cell division after wounding and provide material sources for AR formation. Importantly, the findings of our study have theoretical and practical significance for the clarification of the influencing mechanism of phenolic and flavonoid synthesis in the process of ancient tree cuttings and the value of wounding in promoting the survival rate of difficult-to-root horticultural and woody plant cuttings.

## Data availability statement

The original contributions presented in the study are included in the article/**Supplementary Material**. Further inquiries can be directed to the corresponding author/s. All mass spectrometry proteomics data raw data are deposited in the iProX database (the dataset identifier PXD042637).

## Author contributions

EC, JL and ZeJ planned and designed the research and managed the project. JL, EC, WG, YD and ZiJ contributed to sample preparation and sequencing. WG and XZ analyzed the data. EC and JZ analyzed the data and wrote the manuscript. WG, ZiJ, LZ and ZeJ revised the manuscript, provided advice on the experiments, and finalized the manuscript. All authors contributed to the article and approved the submitted version.
