# Supplementary material for: Metabolic profiling reveals key metabolites regulating adventitious root formation in ancient Platycladus orientalis cuttings
Source: Front Plant Sci. 2023 Jul 11;14:1192371. doi: 10.3389/fpls.2023.1192371 (PMC10367097; doi:10.3389/fpls.2023.1192371)
Supplement: Supplementary file 1 [file DataSheet_1.pdf]

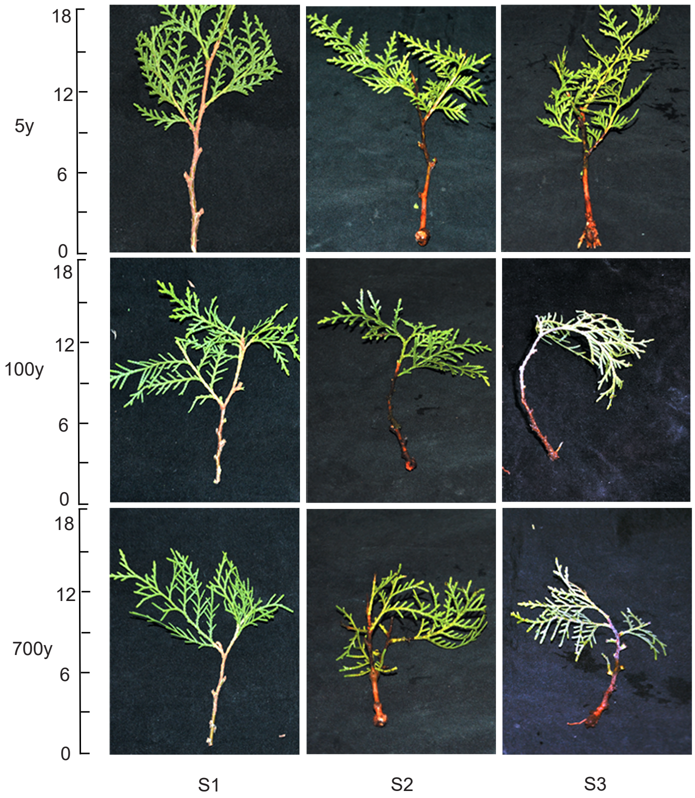

Figure S1 Morphological characteristics of *P. orientalis* cuttings during AR formation. Stages 1, 2, and 3 refer to 0, 45, and 90 days after cuttings were removed from trees, respectively. Scale bar in centimeters.
